# Homogenization of Mammalian Tissues

**DOI:** 10.1100/tsw.2002.849

**Published:** 2002-06-13

**Authors:** John Graham

**Affiliations:** School of Biomolecular Sciences, Liverpool John Moores University, UK

**Keywords:** homogenization, liver, brain, skeletal muscle, nuclei, mitochondria, subcellular organelles, subcellular membranes

## Abstract

Satisfactory homogenization of a tissue is a necessary prerequisite to any fractionation schedule. A detailed protocol is given for rat liver because of the widespread use of this tissue. Although this technique should be broadly applicable to any soft tissue and to any subsequent fractionation procedure, there are certain tissues and applications that require either minor or extensive modification. Some of these points are addressed in the Notes section.

**METHOD TYPE:** extraction, isolation, purification and separation

**SUB METHOD TYPE:** centrifugation

## INTRODUCTION

Mammalian tissues fall generally into two groups: soft tissues (e.g., rat liver) and hard tissues (e.g., bovine muscle) and routinely the types of homogenizer used to disrupt these tissues are liquid shear (Potter-Elvehjem or Dounce) or mechanical shear (e.g., Polytron), respectively. The situation is not clear cut however since hard tissues may be rendered susceptible to liquid shear homogenization by treatment with hydrolytic enzymes.

Whatever technique is used, it is good practice to facilitate the homogenization by an initial coarse mincing of the tissue with scissors, scalpels, or (for large masses of tissue) a mincer.

Highly vascular tissues such as rat liver may require some form of perfusion to remove blood from the vasculature prior to homogenization. This is particularly true if the nuclear pellet is to be processed, for any erythrocytes in the homogenate will sediment at low g-forces. Erythrocytes may also interfere with the functional characterization of a particular organelle, for example the catalase in these cells may obscure any assessment of the fractionation of peroxisomes by measurements of this enzyme. Perfusion can be carried out after sacrificing the animal, simply by injection of buffered saline or homogenization medium through the portal vein after cutting the blood vessels above the liver. It is best carried out however under anaesthesia when the portal vein can be properly cannulated. This must be performed by a trained and licensed operative. Stirring the coarse mince produced prior to homogenization, in medium; allowing the tissue pieces to settle out and decanting the liquid can also remove some of the blood. This process can be repeated.

It should of course always be borne in mind that homogenization of any tissue in any aqueous medium is bound to disturb the delicately balanced functions of the intact cells and to study any one subcellular compartment in the absence of others is an artificial situation. Nevertheless, as evinced by the plethora of scientific papers, the strategy of homogenization in an aqueous medium, followed by fractionation, is widely used to study subcellular processes. Due attention must be paid to detrimental effects of degradative enzymes that might be released from organelles during the homogenization and subsequent procedures. Some of these problems are addressed in Notes 1 and 2.

## HOMOGENIZATION OF RAT LIVER

### Material and Equipment

Homogenization medium (HM): 0.25 M sucrose, 1 mM EDTA, 10 mM HEPES-NaOH, pH 7.4 (see Notes 1–5)

Potter-Elvehjem homogenizer (30–40 ml), clearance approx 0.08 mm (see Note 6)
Wall mounted, high-torque, thyristor-controlled electric motor
Muslin or nylon mesh (75-µm pore size)

### Method (see Note 7)

1. Keep all the equipment on ice and carry out all operations at 0–4°C.
2. Perfuse the liver if necessary, then rapidly excise the tissue into ice-cold HM.
3. Transfer the liver into a 50-ml beaker (on ice) and mince with scissors, the pieces of tissue should be no more than 30 $mm^3$.
4. For one liver (10–12 g) suspend the coarse mince in 40 ml HM. Stir and then decant the liquid after the mince has settled out. Repeat this process and finally suspend in 40 ml of HM.
5. Secure the ice-cold pestle in the chuck of the electric motor.
6. Suspend the liver pieces in the medium by agitation and rapidly transfer half to the glass vessel of the homogenizer.
7. With the pestle rotating at 500–800 rpm homogenize the liver using 5–6 up-and-down strokes of the pestle. If the tissue becomes compacted at the bottom of the vessel, withdraw the pestle and allow the vortex action in the liquid to resuspend the tissue.
8. When all the pieces of tissue have been homogenized, repeat the procedure with second half of the tissue suspension.
9. If required, filter through nylon gauze or three layers of muslin to remove undisrupted cells and connective tissue. Do not force the suspension through the filter by squeezing.

## NOTES

1. It is common to guard against possible protein hydrolysis in the homogenate by including a cocktail of protease inhibitors in HM: for example, 1 mM phenylmethylsulphonyl fluoride (PMSF) and 2 μg/ml each of antipain, leupeptin, and aprotinin. These however are not effective against the activity of glycosidases or lipoxygenases. The latter are instrumental in the production of potentially damaging lipid hydroperoxides. See Ref. [1].
2. In certain cases it may be possible to use a nonaqueous homogenization medium that will prevent the removal of surface proteins from organelles and the solubilization of certain enzymes. Ethylene glycol and formamide have been used successfully for the isolation of nuclei[2] and minimize loss of certain RNA polymerases but the isolation of other organelles is less well established and, moreover, is compromised by the subsequent requirement to assay enzymes. More often than not, nonaqueous media pose more problems than they solve.
3. Routinely, most **soft tissues** are homogenized in 0.25 M sucrose, buffered with low concentrations of an organic buffer such as Tris, Hepes, or Tricine at a pH between 7 and 8. Often 1 mM EDTA is included to reduce aggregation, but if the organelle of interest is the nucleus, the EDTA is replaced with 25 mM KCl and 5 mM $MgCl_2$, while for sheets of plasma membrane use 1 mM $MgCl_2$. For mitochondria, the sucrose may be replaced by mannitol and for peroxisomes 0.1% ethanol is included.
4. **Brain** tissues are frequently disrupted in 0.32 M sucrose rather than 0.25 M. Hypo-osmotic media (e.g., 10 mM Tris-HCl, pH 7.5 or 5 mM EDTA, pH 7.4) are often used with **intestinal mucosa**.
5. Media for **muscle** homogenization are also quite variable and although compositions not unlike those for soft tissues have been used, KCl is often included (up to 180 mM) to solubilize some of the protein and prevent the formation of gels. The following media have been successfully used: 0.21 M mannitol, 70 mM sucrose, 0.1 mM EDTA, 0.5% bovine serum albumin (BSA), 10 mM Tris-HCl, pH 7.4 or 0.1 M sucrose, 10 mM EDTA, 46 mM KCl, 0.5% BSA, 100 mM Tris-HCl, pH 7.4[3]. After coarse mincing of muscle tissue, it is commonly softened by incubating with Nagarse at 5–50 mg/100 ml at $4^{o}$C for about 5 min.
6. To isolate sheets of plasma membrane it may be preferable to replace the Potter-Elvehjem homogenizer with a loose-fitting Dounce homogenizer (clearance 0.1–0.3 mm) using about 10 strokes of the pestle and filter before processing further.
7. A useful general introduction to homogenization methods is given in Ref. [4].

## ACKNOWLEDGEMENTS

The author and TheScientificWorld wish to thank Axis-Shield PoC, AS, Oslo, Norway for their kind permission to adapt OptiPrep™ Application Sheet S5 in the preparation of this Protocol Article.
